# Mrs. Humphry Ward

**Published:** 1920-04-03

**Authors:** 


### THE PUBLIC WELL-BEING.

### MRS. HUMPHRY WARD.

### A Pioneer in Children's Service.

Mrs. Humphry Ward's death last week not only brought to an end the series of books which were still bought in numbers by the libraries, it has deprived the Children's Play Centre Association of its President, and the Women's Parliamentary Advisory Council of its Hon. Treasurer, and it has removed the name of a woman from the list of Justices of the Peace.

As a novelist Mrs. Ward suffered in her later years from the overwhelming success of her earlier books. One detects in newspaper memoirs an apologetic note as if the writers distrusted the judgment which had made them so keenly interested in "Robert Elsmere," "Marcella," and "David Grieve." But the truth is that Mrs. Ward chose her subjects admirably, she knew how to describe a scene and create an atmosphere, and though once the topical interest of the argument was gone the argument seemed over laboured, she could tell a story well. Her readers felt that they were introduced to the intricacies of the political and literary world by one who was entirely at home there, and it was an added pleasure to believe that they could identify among her characters real personalities. These were the things that accounted for her immense popularity with American and Colonial readers. She showed them the England they most wanted to know.

It seemed strange to some people that a woman who was herself so interested in practical politics, and who wrote so much about the influence of women in the political world, should have taken the attitude she did on the suffrage question. To leading feminists who sincerely admired her intellectual powers and her social work it was a lasting regret that she would not join them. To the anti-suffrage party she was a tower of strength, because they could always point to her as the most distinguished literary woman of the day. She was always an honourable opponent, and, when fighting strenuously and rather pathetically to the end, she had to accept defeat, she accepted it with dignity.

To suffragists the scheme Mrs. Ward propounded in 1913 for setting up a Council of women experienced in social work, to act as advisers to members of Parliament in preparing legislation, appeared fantastic and illogical, but it was carried through. The public has just learned for the first time that through the intervening years a Council of representative women has been collecting and sifting information and securing the passing of amendments to many Bills. Mrs. Ward, who was Hon. Treasurer of this Council, did a great deal of work on educational questions. "She was invaluable," says one of her colleagues. "She had a most comprehensive grasp of her subjects, and when one took a difficulty to her it was extraordinary how quickly she would seize the point and find a solution. She was rigidly accurate, and no half-fact brought in by our inquiries ever satisfied her. She would refer it back for full information. Nothing, I believe, ever gave her greater pleasure than the carrying of the amendment in the Education Act making it compulsory for local authorities to provide physically defective children with educational facilities. That amendment was largely her work."

It will be remembered that Mrs. Ward initiated the first invalid school. Those delicate children who travel to and from school in ambulances, and who ultimately in many cases are able largely to overcome their disability, owe their happiness primarily to her.

It was on Mrs. Ward's initiative also that the Passmore Edwards Settlement was founded in Bloomsbury twenty-six years ago. This became a women's settlement in 1916, and during all those years she was its President. This settlement was the headquarters of the Children's Play Centre Movement, which has spread over all the poorer parts of London—where there are now thirty-three centres—and to the large towns. The idea was that out of school hours the school buildings and playgrounds should still benefit the children who had nowhere to play but in the streets, and who were often not able to get into their own homes until the mothers returned from work. In three centres games were organised, the children were taught to draw and paint, to make simple articles, and dance and sing, and when possible children's libraries were formed. It was always delightful to visit one of these centres, such as that in St. George's-in-the-East, and see how happy the children were.

Extending this scheme in all directions, Mrs. Ward improved on it some years ago when she started the first vacation schools, and provided the same amusement for children during the long holidays. Only those familiar with the congested districts of our great towns can realise how well Mrs. Humphry Ward has served the children.

